# 3D mapping of elastic modulus using shear wave optical micro-elastography

**DOI:** 10.1038/srep35499

**Published:** 2016-10-20

**Authors:** Jiang Zhu, Li Qi, Yusi Miao, Teng Ma, Cuixia Dai, Yueqiao Qu, Youmin He, Yiwei Gao, Qifa Zhou, Zhongping Chen

**Affiliations:** 1 Beckman Laser Institute, University of California, Irvine, Irvine, California 92612, USA; 2 Department of Biomedical Engineering, University of California, Irvine, Irvine, California 92697, USA; 3 Department of Biomedical Engineering, University of Southern California, Los Angeles, California 90089, USA; 4 College of Science, Shanghai Institute of Technology, Shanghai 201418, China

## Abstract

Elastography provides a powerful tool for histopathological identification and clinical diagnosis based on information from tissue stiffness. Benefiting from high resolution, three-dimensional (3D), and noninvasive optical coherence tomography (OCT), optical micro-elastography has the ability to determine elastic properties with a resolution of ~10 μm in a 3D specimen. The shear wave velocity measurement can be used to quantify the elastic modulus. However, in current methods, shear waves are measured near the surface with an interference of surface waves. In this study, we developed acoustic radiation force (ARF) orthogonal excitation optical coherence elastography (ARFOE-OCE) to visualize shear waves in 3D. This method uses acoustic force perpendicular to the OCT beam to excite shear waves in internal specimens and uses Doppler variance method to visualize shear wave propagation in 3D. The measured propagation of shear waves agrees well with the simulation results obtained from finite element analysis (FEA). Orthogonal acoustic excitation allows this method to measure the shear modulus in a deeper specimen which extends the elasticity measurement range beyond the OCT imaging depth. The results show that the ARFOE-OCE system has the ability to noninvasively determine the 3D elastic map.

Elastography is applied to clinical diagnosis based on the measurement of biomechanical properties which may be affected by the tissue structure and be related to histopathological changes. A typical elasticity imaging method includes three essential steps: excitation for inducing biomechanical vibration, measurement of sample response after excitation, and estimation of elastic parameters^1^. Compared with magnetic resonance measurements^2^ and ultrasound measurements^3^, OCT measurements have much higher spatial resolution, which is usually ~10 μm; thus optical coherence elastography (OCE) can provide elastic properties of tissues with more spatial details^4^,^5^,^6^,^7^. Recently, benefiting from the ultrahigh-resolution of optical coherence microscopy, ultrahigh-resolution elastic measurements have been obtained using OCE^8^.

In order to induce the sample vibration, several methods has been reported: a mechanical actuator induces displacement of the specimen surface with contact between the specimen and the actuator-tip^9^, an air-puff device applies a force on the specimen surface through an output stream of low-pressure air^10^,^11^, and a laser device generates a heating and cooling process that results in a surface acoustic wave^12^. However, vibration is not easily induced deep within the specimen. Magnetic nanoparticles embedded within the specimen can be driven by the magnetic field and displace the internal medium^13^, and a needle tip inserted inside the specimen causes internal deformations^14^, which may damage the specimen and be inconvenient in some clinical applications. Acoustic force generated by an ultrasonic transducer can noninvasively excite internal tissues without contact; thus, it is suitable for inducing mechanical vibration in deeper tissue^15^. Benefiting from the approximate uniformity of ARF along the ultrasound axial direction, ARF can induce simultaneous vibration of internal tissues in a range of several millimeters without movement of the ultrasound transducer and use of an array transducer, thus reducing the system complexity.

For determination of elastic properties using optical elastography, several methods have been developed. The axial displacement measurement can provide local strain rates related to elastic property^16^,^17^, evaluate elastic properties by solving the wave equations^18^, or compare the elastic moduli according to vibration amplitude^19^. The measurement of resonant frequency in response to an external force provides quantitative assessment of the elastic modulus in tissues^20^. The surface wave method utilizes frequency-dependent phase velocity of the surface acoustic wave to evaluate the Young’s modulus on the tissue surface^21^, but the detection depth is limited. Shear wave measurement, unlike other methods, enables the quantitative determination of shear modulus in a deeper tissue by simple calculation^22^, and the values obtained from the shear wave measurement are comparable among different samples and experiments.

In the shear wave measurement, the wave propagation induced by ARF is visualized by the Doppler OCT phase method under parallel excitation^23^. The ultrasound transducer can be located on the opposite side of the OCT detection lens^23^, which may be inconvenient in clinical applications, or be located on the same side of the OCT lens^24^, which requires a ring transducer to co-align the ultrasound and OCT beams. The mapping of the biomechanical properties of tissue is limited to OCT imaging depth. To overcome these limitations, we previously developed an ARFOE-OCE system using the Doppler variance method where the acoustic force is perpendicular to the OCT detection beam^15^. We demonstrated that the shear modulus of a sample as deep as at least 7.5 mm, which is beyond the OCT detection range, can be quantified. However, only one-dimensional (1D) shear wave propagating along the OCT beam was detected, and the map of the shear modulus in a two-dimensional (2D) plane requires point-by-point scans of the samples.

In this study, we constructed a 3D map of the shear modulus with high resolution by recording the 3D shear wave propagation. Unlike previous 3D optical elastography, which measures the strain rate^16^,^25^, our method can non-invasively and quantitatively measure the shear modulus inside the specimen. A homogeneous agar phantom and a two-layer agar phantom were tested using the ARFOE-OCE system. The shear wave propagations were compared with those from finite element modeling, and the shear moduli were compared with those from mechanical tests. The results show that this system has the ability to determine a 3D map of the shear modulus in a deeper phantom with a simpler system setup and data processing.

## Visualization of the shear wave using ARFOE-OCE

**Figure 1:**
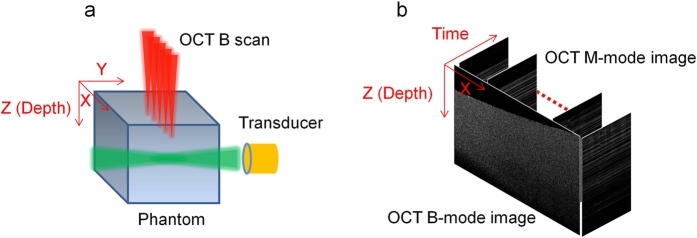
Data processing for 3D shear modulus mapping. (**a**) Schematic of the ARF excitation and OCT detection. The acoustic force is parallel to the Y axis, OCT beam is parallel to the Z axis, and the OCT B-scan is performed along the X axis. (**b**) OCT M-mode images and the constructed B-mode image during the measurement.

The shear wave induced by the ARF is detected by the Doppler OCT variance method. The vibration generated by the acoustic force is perpendicular to the OCT detection direction, and the shear wave propagates from the ARF focus to the surrounding medium. The relative position of the OCT beam and the ultrasound transducer is shown in Fig. 1(a), where the acoustic force is parallel to the Y axis and the OCT beam is parallel to the Z axis. In order to visualize the shear wave propagation in a 2D plane (X-Z plane) perpendicular to the acoustic axial direction, each A-line consists of 512 pixels and the pixel size along the Z axis is 9.8 μm/pixel; 500 A-lines are captured for each M-scan at each position, corresponding to a capture time of 10 ms; 3200 positions are imaged for each B-scan along the X axis with a range of 8.3 mm and the image pixel is adjusted to match a pixel size of 9.8 μm. The ultrasonic force is applied to the specimen for 0.4 ms when the OCT beam is located at each position. Then the Doppler variance method, which is sensitive to the displacement perpendicular to the OCT beam direction, is applied to the 500 A-lines for visualization of vibration at each position. The intensity-based Doppler variance is calculated by the following equation^26^,^27^:


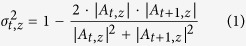


where *A*_*t,z*_ and 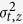 are the complex data and Doppler variance at the A-line of time *t* and the depth of *z*.

After each B-scan, a cross-sectional image in the X-Z plane is constructed by time, as shown in Fig. 1(b). The shear wave propagation in the 2D plane can be visualized over time, and thus, the shear wave velocity 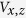 can be measured. Then the shear modulus 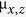 is calculated by the following equation^22^:


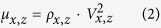


where 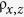 is the density of the specimen.

By changing the B-scan position along the Y direction using the galvanometer, we repeat the above M-scans and B-scan on each plane. Due to the uniformity of the acoustic force along the Y direction near the focus, a C-scan along the Y direction is performed in a 2.0 mm region with a step size of 135 μm, and then a cubic spline interpolation method is utilized to construct data points with a step size of 9.8 μm. With a series of 2D maps at different Y positions, a 3D map is constructed.

### Propagation of the shear wave in a homogeneous phantom

The ultrasound transducer generates an acoustic force orthogonal to the OCT detection direction and induces a vibration perpendicular to the OCT beam. The vibration propagates from the ARF focus to the surrounding medium, and the propagation direction is perpendicular to the vibration direction so that the transverse wave propagating inside the medium is predominantly a shear wave. A compressional wave cannot be detected by this system due to its much higher velocity.

**Figure 2:**
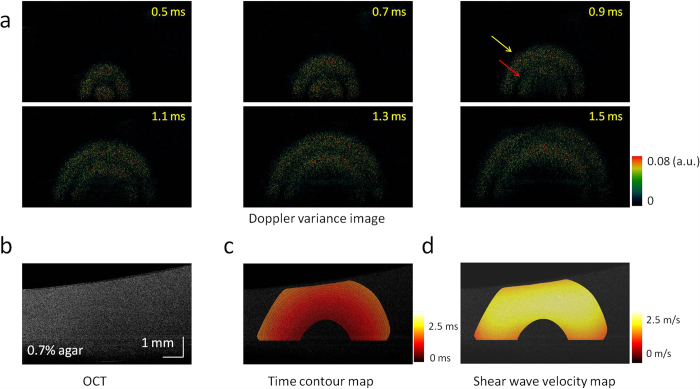
2D visualization of the shear wave propagation and mapping of the shear wave velocity in a homogeneous phantom with 0.7% agar. (**a**) Doppler variance images during the shear wave propagation. (**b**) OCT image of the homogeneous phantom. (**c**) Time contour map of the shear wave propagation. (**d**) 2D map of the shear wave velocity. The shear wave velocity is almost uniform in the homogeneous phantom.

The shear wave propagation in a 0.7% agar homogeneous phantom is shown in Fig. 2(a), and the OCT image of this phantom is shown in Fig. 2(b). Supplemental Video 1 shows the shear wave propagation at a time interval of 0.02 ms, including 0.4 ms of ARF excitation at the beginning. As the Doppler variance method only detects the amplitude of displacement without consideration of the direction, stronger signals indicate faster displacements during the shear wave propagation. With the process of time, there were two strong vibrations propagating from the ARF focus with uniform velocity. The first vibration was induced at the beginning of ARF excitation [yellow arrow in Fig. 2(a)], and the second one was induced at the end of ARF excitation [red arrow in Fig. 2(a)]. The shear wave velocity was calculated after stopping the ARF excitation in order to eliminate distortion due to the vibration during ARF excitation. The time of the first shear wave arriving at a measured point was recorded, and the time contour map of the shear wave propagation was measured, as shown in Fig. 2(c). The distances between adjacent contours were approximately the same, which indicates that the shear wave propagates at an almost uniform velocity. The direction of the wave propagation at each point can be determined by a gradient calculation on the time contour map, and then the shear wave velocity can be calculated by a ratio of the distance along the propagation direction to the traveling time at each point, which is shown in Fig. 2(d). From Fig. 2(d), the velocity of shear wave in the 2D plane is almost uniform, and the velocity is 1.9 ± 0.3 m/s. The shear wave velocity is almost uniform in the homogeneous phantom.

### Propagation of the shear wave in a two-layer phantom

**Figure 3:**
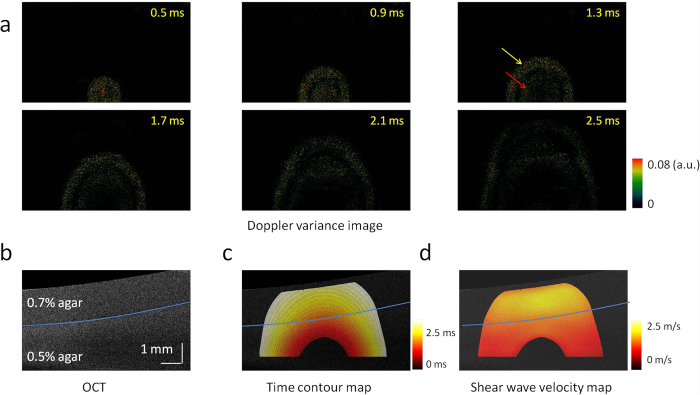
2D visualization of the shear wave propagation and mapping of the shear wave velocity in a two-layer phantom with 0.7% agar in the top and 0.5% agar in the bottom. (**a**) Doppler variance images during shear wave propagation. (**b**) OCT image of the two-layer phantom. There are no obvious differences between the two layers. (**c**) Time contour map of the shear wave propagation. (**d**) 2D map of the shear wave velocity. The shear wave velocity changes significantly when the wave travels from the bottom layer to the top layer.

To verify this system for the measurement of a heterogeneous specimen, we imaged a two-layer phantom with 0.7% agar in the top and 0.5% agar in the bottom. The shear wave propagation visualized by the Doppler variance method is shown in Fig. 3(a), and the OCT image is shown in Fig. 3(b). Supplemental Video 2 shows the shear wave propagation over time, including 0.4 ms of ARF excitation at the beginning. From the OCT image, there are no obvious differences between the two layers. However, the propagation of the shear wave accelerates immediately after the wave travels through the boundary from the soft layer to the stiff layer. Similar to the shear wave propagation in the homogeneous phantom, two strong vibrations were recorded at the beginning of ARF excitation [yellow arrow in Fig. 3(a)] and at the end of ARF excitation [red arrow in Fig. 3(a)], respectively. To remove the interaction of the two vibrations and the distortion during the ARF excitation, we measured the time contour map by tracking the first vibration propagation after cessation of ARF excitation, as shown in Fig. 3(c). The distance between the two adjacent contours becomes longer, and thus, the velocity of the shear wave is higher in the top layer than in the bottom layer. After determination of the wave propagation direction at each point on the time contour map, the wave velocity was mapped in a 2D plane, as shown in Fig. 3(d). Though no obvious differences are observed in the OCT image, the shear wave velocity changes significantly from 1.0 ± 0.2 m/s to 1.8 ± 0.1 m/s when the wave travels from the 0.5% agar phantom to the 0.7% agar phantom.

### 3D visualization of the shear wave propagation

**Figure 4:**
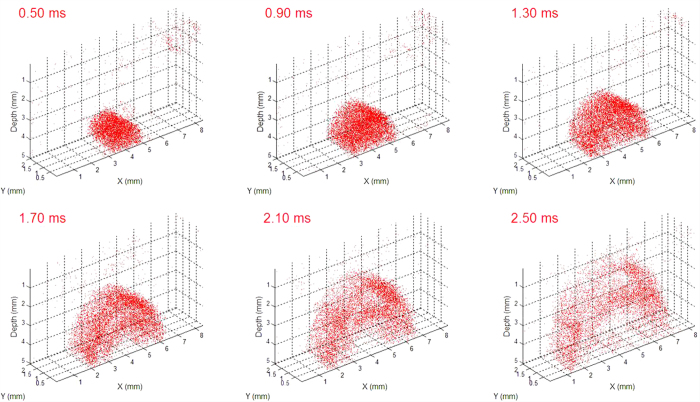
3D visualization of shear wave propagation in a two-layer phantom with 0.7% agar in the top and 0.5% agar in the bottom. ARF excitation induces almost uniform vibration along the acoustic force in a range of 2 mm.

**Figure 5:**
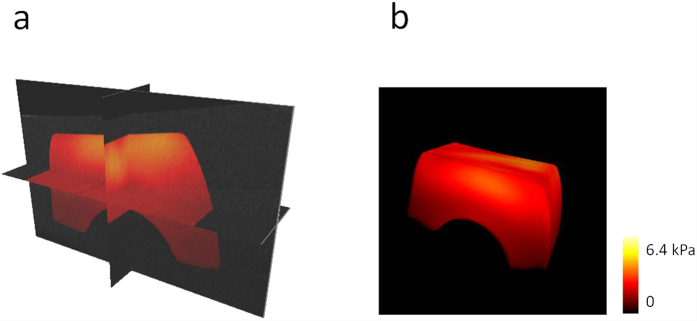
3D map of the shear modulus in a two-layer phantom. **(a)** Orthoslice. **(b)** Volume rendering. Obvious differences of the shear modulus can be measured between the top layer with 0.7% agar and the bottom layer with 0.5% agar. Higher shear modulus in the top layer indicates a higher stiffness.

Due to the ultrasonic force uniformity along the axial direction, ARF induces simultaneous vibrations along the axial direction of the transducer in a range of ~2.0 mm in our system. The shear wave propagation can be visualized in a series of planes perpendicular to the axial direction of the transducer using an OCT C-scan so a series of 2D maps of the shear wave velocity can be measured using this system without moving the ultrasound transducer. The 3D shear wave propagation can be constructed from these 2D data. Figure 4 shows the 3D shear wave propagation in a two-layer phantom. ARF excitation induces almost uniform vibration along the transducer axial direction and the propagation of the waves in different planes has similar patterns. Supplemental Video 3 shows the 3D shear wave propagation at a time interval of 0.02 ms, including 0.4 ms of ARF excitation at the beginning. After analysis of shear wave velocity on each plane, a 3D map of the shear wave velocity can be calculated and then the 3D map of the shear modulus can be obtained from Equation 2. The 3D map of the shear modulus in a two-layer phantom is shown in Fig. 5. From the orthoslice in Fig. 5(a) and the volume rendering in Fig. 5(b), there are obvious differences of the shear moduli between the top layer with 0.5% agar and the bottom layer with 0.7% agar. Supplemental Video 4 shows the animation of the 3D map of the shear modulus with 360° rotation. The shear modulus is measured to be 2.71 ± 0.12 kPa for the top layer and 1.09 ± 0.04 kPa for the bottom layer using the ARFOE-OCE system. The elastic modulus is also measured using a mechanical test system, and the shear modulus is 2.59 ± 0.16 kPa for a homogeneous 0.7% agar phantom and 0.98 ± 0.13 kPa for a homogeneous 0.5% agar phantom, respectively. There are differences in shear moduli between the OCE measurements and the mechanical test results partially because OCE was used to detect the surface portion of a sample with a depth of ~5 mm while the mechanical tests detected the sample with a depth of ~10 mm in our experiments.

### Simulation of shear wave propagation

**Figure 6:**
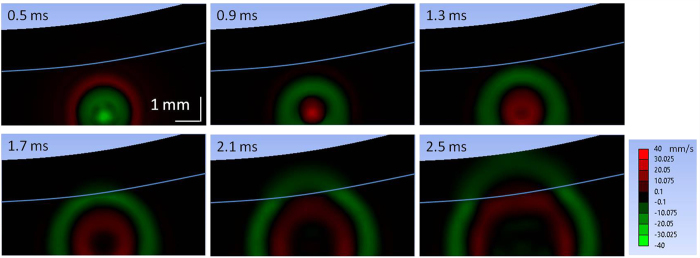
FEA simulation of shear wave propagation in a two-layer phantom with 0.7% agar in the top and 0.5% agar in the bottom. White line indicates the boundary of two layers. The color bar indicates the local velocity in lateral direction.

Based on the elastic moduli measured using the ARFOE-OCE system from Fig. 3, the propagation of shear wave was simulated by FEA in a two-layer phantom with 0.7% agar on the top and 0.5% agar on the bottom. In this simulation, the acoustic excitation area was determined by the pressure profile of the ultrasound transducer, and the excitation location was the same as the one determined from experimental observations in Fig. 3(a). The excitation force in the simulation was set so that the maximum displacement of the phantom in the lateral direction matched the one obtained from the experiment. The agar phantom was perturbed by a constant pressure of 0.4 ms duration at the beginning. Propagation of the shear wave was tracked after removal of acoustic pressure. The velocities of vibration for each time point were exported from ANSYS. The shear wave propagation in a two-layer phantom is shown in Fig. 6.

The propagation visualization of shear waves in our measurement agrees well with the simulation using FEA. In the FEA simulation, two opposite vibrations propagate from the ARF focus. The first vibration (green wave) due to the acoustic force is generated during ARF excitation and the second (red wave) due to the recovery of elastic deformation is generated after stop of ARF excitation. When the wave travels through the boundary from the bottom layer to the top layer, shear wave propagation accelerates immediately in the simulation. The simulated wave patterns are similar to those obtained from the experiment.

## Discussion

Using the ARFOE-OCE system, the 3D map of the shear modulus was visualized based on the OCT Doppler variance method. The OCT imaging covers a range of 8.3 mm × 2.0 mm × 5.0 mm in the X, Y and depth direction. The OCT imaging ranging along the X and Y direction can further be extended by increasing the scanning range of the galvo mirror, and the imaging depth can be determined by the optical properties of the specimen and the OCT system, especially the swept source. However, the ranges of detection mainly depend on the vibration intensity in the specimen, which can be affected by the power and depth of focus of the ultrasonic force, and the attenuation of the ultrasound by the specimen. Typically, an unfocused transducer will lead to a longer range of uniform ARF and, thus, a longer detection range along the transducer axial direction. The minimum ARF power required is determined by the sensitivity of the OCT Doppler variance method.

As the shear wave velocity is calculated by the ratio of the traveling distance to the time interval between adjacent frames, the spatial resolution of the shear modulus map using the ARFOE-OCE system depends on the spatial resolution of the OCT system and the A-line speed. The spatial resolution of the shear modulus map is determined by the larger value between the OCT imaging resolution ΔD and the velocity mapping resolution *V*_*x,z*_/*f*, where *V*_*x,z*_ is the shear wave velocity and *f* is the frame rate. Typically, a higher optical resolution and a faster A-line speed result in a higher spatial resolution of the shear modulus map. In this measurement, the axial and lateral resolutions of the OCT are 7.6 μm and 17.7 μm, respectively. The frame rate is 50 kHz, and the velocity mapping resolution is 20.0 μm when the shear wave velocity is 1.0 m/s. So the maximum resolution of the shear modulus map is 20.0 μm. Using optical coherence microscopy, OCE has recently achieved a spatial resolution (x, y, z) of 2 × 2 × 16 μm for strain estimation without the values of elastic moduli^8^.

The vibration induced by ARF is analyzed by a Doppler variance method, which is sensitive to transverse displacement^26^. As the vibration direction is perpendicular to the OCT beam in this measurement, the Doppler variance method may provide better visualization of waves than other OCT methods. The SNR of an OCT system, the analysis method of OCT data, and the recognition method of waves may affect the mapping of the time contour of the shear wave propagation.

The shear modulus was calculated after determination of the density and the shear wave velocity. The error from the density estimation will result in the same percentage of error for the calculation of shear modulus. The densities of most tissues have been studied in previous publications^28^,^29^. If the density cannot be determined easily, we can directly compare the square of the shear wave velocity to distinguish the stiffness when the density does not change significantly.

The ARFOE-OCE system has the ability to detect the shear modulus beyond the OCT imaging depth. If the ultrasound transducer located under the OCT imaging depth is moved downwards to a known step, the shear wave velocity can be measured by the ratio of the step distance of the ARF focus to the time change of the shear wave arriving at the same depth. Based on this principle, the coagulation process of whole blood can be monitored by ARFOE-OCE, even though the OCT imaging depth is limited near the surface of highly scattering whole blood^30^. Combining the 3D mapping of the shear modulus with its ability for the detection beyond the OCT imaging depth, ARFOE-OCE system has the potential to detect the elastic properties in a much wider range of the tissue.

The upper limit of the detectable shear modulus *M*_*max*_ can be determined by the maximum OCT imaging depth *D* in the sample and the frame rate *f*, which is described as 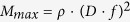. If *ρ* is 1 g/cm^3^, *D* is 5 mm and *f* is 50 kHz, the theoretical maximum detectable modulus is 62.5 MPa. The lower limit of the detectable shear modulus *M*_*min*_ can be determined by the OCT resolution ΔD and the frame rate *f*, which is described as 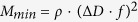. If *ρ* is 1 g/cm^3^, ΔD is 10 μm and *f* is 50 kHz, the theoretical minimum detectable modulus is 0.25 kPa. When we decrease the frame rate, the lower limit will decrease. The measurement range of our system can cover the elasticity of most soft biological tissues^31^,^32^, as well as monitor the blood coagulation^30^.

For 2D shear modulus mapping, 500 A-lines are captured for each M-scan at each position, corresponding to the capture time of 10 ms, and 3200 positions are imaged for each B-scan along the X axis, which means that the total acquisition time of the M-B scan is 32 s when a 50 kHz swept source is used. The acquisition time can be significantly shortened if we capture a B scan using a higher A-line rate swept source. Recently, the swept source with an A-line rate of 1.5 MHz has been used for high speed OCE based on B scan capture^33^. High speed OCE will shorten the acquisition time, which is important for clinical applications.

The ARFOE-OCE system uses ultrasonic force to induce a shear wave and measures the shear modulus by tracking the shear wave propagation. Due to the highly scattering properties of tissues, the detection depth of OCT may be limited, which will result in a limited range detection of the shear wave. The ultrasound should also be coupled to the sample by gel or water. However, ARFOE-OCE has provided new opportunities for the system design. Using the orthogonal ARF excitation system, the ultrasonic wave could conveniently reach the ocular target through the eyelid or the outer corner of the eye. This system has also been successfully used for blood coagulation monitoring^30^. The ARFOE-OCE system has the potential to perform *in vivo* imaging because of its noninvasive nature and the possibility to shorten acquisition time.

## Conclusions

We have developed an ARFOE-OCE system for 3D mapping of the shear modulus in tissue-equivalent agar phantoms. The wave propagation induced by ARF is visualized by high-resolution noninvasive OCT Doppler variance imaging in three dimensions. This method gives a quantitative measurement of the shear modulus in the deep range of a specimen using orthogonal ARF excitation, and the excitation unit of this system is simplified for 3D mapping of the shear modulus without the need of transducer movement. The 3D mapping of the shear modulus in a two-layer phantom shows that this system has the ability to noninvasively quantify the elastic properties in 3D specimens using a simpler system setup and data processing.

## Methods

### Experimental setup

**Figure 7:**
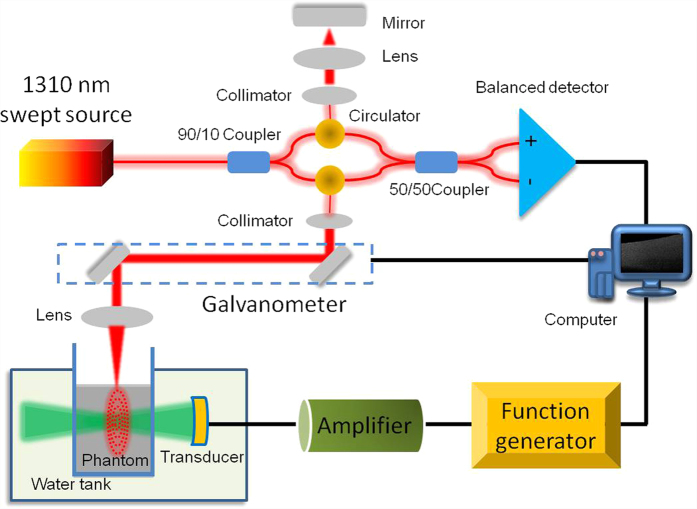
The ARFOE-OCE system based on a swept source OCT unit and acoustic radiation force excitation unit. The axial direction of the ultrasound transducer is orthogonal to the OCT detection direction. ARF-induced vibration is perpendicular to the OCT detection direction and propagates from the ARF focus to the surrounding medium.

The ARFOE-OCE system is based on a swept source OCT unit and ultrasonic radiation force excitation unit, which is shown in Fig. 7. The light from the swept source with a central wavelength of 1310 nm and an A-line speed of 50 kHz is split 90% into the sample arm and 10% into the reference arm. In the sample arm, the light reaches the sample through a collimator, a galvanometer for two-dimensional light scanning, and a scan lens with a focusing length of 36 mm. In the reference arm, the light is reflected by a mirror after passing through a collimator and a focusing lens. An ultrasound transducer is driven by the sine wave with a frequency of 4.5 MHz, peak-to-peak voltage of 160 V, and duration time of 0.4 ms during each OCT M-scan. The axial full width at half-maximum power (FWHM) is 7.9 mm, and the acoustic force is almost uniform in a range of ~2.0 mm at the focus. The pressure profile of the ultrasound transducer is shown in Supplemental Fig. 1. The axial direction of the ultrasound transducer is orthogonal to the OCT beam direction. The tissue-equivalent phantom is placed in a container with a film window passable for the ultrasound. The box and the transducer are immersed in water. In order to avoid the strong reflection from the surface of a phantom, the surface is not perpendicular to the OCT beam when we place the phantom.

### Phantom preparation

The phantoms are made of agar with concentrations of 0.5% and 0.7% w/v for different stiffnesses. For construction of a phantom, the granulated agar is dissolved in distilled water at 25 °C. Then the agar solution is heated to 95 °C during stirring. After cessation of heating, the agar solution is cooled to 60 °C with continuous stirring, and then 0.6% v/v intralipid solution is mixed with the agar solution to increase light scattering and ensure a high detection depth. After stirring for 5 min, the final solution is poured into a container and stored in the refrigerator at 4 °C for solidification. The homogeneous phantom contains 0.7% agar, and the two-layer phantom contains 0.7% agar in the top layer and 0.5% agar in the bottom layer. A density of 1.0 g/cm^3^ is used for both the 0.5% agar phantom and the 0.7% agar phantom for the calculation of shear moduli.

### Finite element analysis

Finite element analysis is used to simulate and reconstruct the elastic wave propagation inside the specimens. 3D models based on ARFOE-OCE setup are created using ANSYS transient structural analysis (ANSYS Inc., Pittsburg, PA). The phantom structure in the FEA is designed to be the same as the structure of the phantom in Fig. 3(b) for convenient comparison of the shear wave propagation. The inclination of the top surface of agar phantoms is considered. The bottom and side of the FEA models are fixed. The densities are set to 1.0 g/cm^3^ and the Poisson’s ratios are set to 0.499 for both 0.7% and 0.5% agar phantoms. No viscosity is considered for simplification. Eight-node solid 185 elements are used for the body mesh. The meshing size is 0.16 mm. Nodal force of 0.3 mN is applied to induce the lateral displacement at the beginning. Four hundred time points are recorded with a time interval of 0.01 ms for the total 4.0 ms simulation duration.

## Additional Information

**How to cite this article**: Zhu, J. *et al*. 3D mapping of elastic modulus using shear wave optical micro-elastography. *Sci. Rep.* **6**, 35499; doi: 10.1038/srep35499 (2016).

## Supplementary Material

Supplementary Information

Supplementary Video 1

Supplementary Video 2

Supplementary Video 3

Supplementary Video 4
