# Supplementary material for: 3D mapping of elastic modulus using shear wave optical micro-elastography
Source: Sci Rep. 2016 Oct 20;6:35499. doi: 10.1038/srep35499 (PMC5071855; doi:10.1038/srep35499)
Supplement: Supplementary Information [file srep35499-s1.doc]

**3D mapping of elastic modulus using shear wave optical micro-elastography**

Jiang Zhu,^1^ Li Qi,^1^ Yusi Miao,^2^ Teng Ma,^3^ Cuixia Dai,^1^ Yueqiao Qu,^1,2^ Youmin He,^1,2^ Yiwei Gao,^2^

Qifa Zhou,^3^ and Zhongping Chen^1,2^*

^1^ Beckman Laser Institute, University of California, Irvine, Irvine, California 92612

^2^ Department of Biomedical Engineering, University of California, Irvine, Irvine, California 92697

^3^ Department of Biomedical Engineering, University of Southern California, Los Angeles, California 90089

*Corresponding author: z2chen@uci.edu


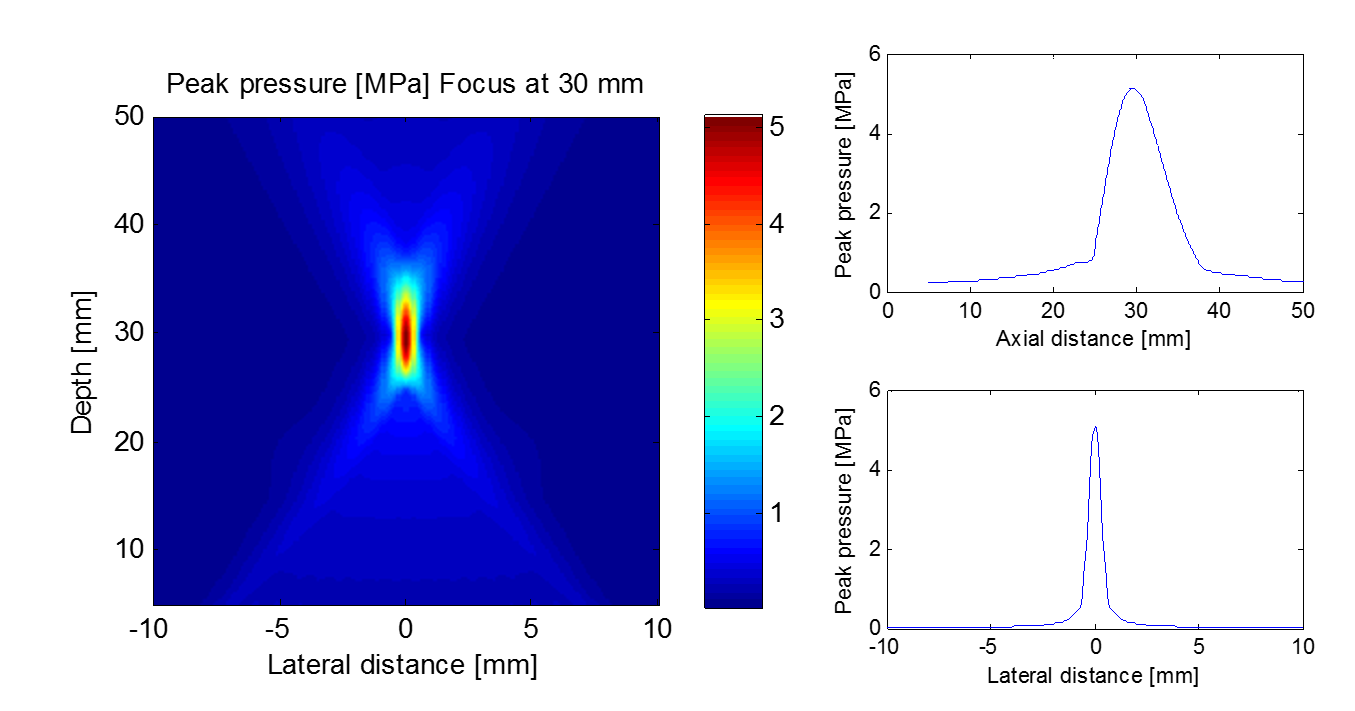


**Supplemental figure 1.** Field II simulation for the pressure profile of ultrasound transducer with a 30 mm focal length. Ultrasound beam creates highly focused force with 7.9 mm full width at half maximum (FWHM) in lateral direction and 0.7 mm FWHM in axial direction.

Supplemental video legends

**Supplemental video 1.** 2D visualization of the shear wave propagation in homogeneous phantom with 0.7% agar. The shear wave velocity is almost uniform in the homogeneous phantom.

**Supplemental video 2.** 2D visualization of the shear wave propagation in two-layer phantom with 0.7% agar in the top and 0.5% agar in the bottom. The shear wave propagation accelerates immediately after the wave travels through the boundary from the soft layer to the stiff layer.

**Supplemental video 3.** 3D visualization of shear wave propagation in a two-layer agar phantom with 0.7% agar in the top and 0.5% agar in the bottom. The propagation of the waves in different planes has similar patterns.

**Supplemental video 4.** Volume rendering with the 360° view for 3D shear modulus mapping in a two-layer phantom with 0.7% agar in the top and 0.5% agar in the bottom.
